# Enhancement of therapeutic potential of mesenchymal stem cell-derived extracellular vesicles

**DOI:** 10.1186/s13287-019-1398-3

**Published:** 2019-09-23

**Authors:** Kyong-Su Park, Elga Bandeira, Ganesh V. Shelke, Cecilia Lässer, Jan Lötvall

**Affiliations:** Krefting Research Centre, Institute of Medicine at Sahlgrenska Academy, University of Gothenburg, Gothenburg, Sweden

**Keywords:** Extracellular vesicles, Exosomes, Mesenchymal stem cells, Immune regulation, Therapeutics

## Abstract

After the initial investigations into applications of mesenchymal stem cells (MSCs) for cell therapy, there was increased interest in their secreted soluble factors. Following studies of MSCs and their secreted factors, extracellular vesicles (EVs) released from MSCs have emerged as a new mode of intercellular crosstalk. MSC-derived EVs have been identified as essential signaling mediators under both physiological and pathological conditions, and they appear to be responsible for many of the therapeutic effects of MSCs. In several in vitro and in vivo models, EVs have been observed to have supportive functions in modulating the immune system, mainly mediated by EV-associated proteins and nucleic acids. Moreover, stimulation of MSCs with biophysical or biochemical cues, including EVs from other cells, has been shown to influence the contents and biological activities of subsequent MSC-derived EVs. This review provides on overview of the contents of MSC-derived EVs in terms of their supportive effects, and it provides different perspectives on the manipulation of MSCs to improve the secretion of EVs and subsequent EV-mediated activities. In this review, we discuss the possibilities for manipulating MSCs for EV-based cell therapy and for using EVs to affect the expression of elements of interest in MSCs. In this way, we provide a clear perspective on the state of the art of EVs in cell therapy focusing on MSCs, and we raise pertinent questions and suggestions for knowledge gaps to be filled.

**Electronic supplementary material:**

The online version of this article (10.1186/s13287-019-1398-3) contains supplementary material, which is available to authorized users.

## Background

Extracellular vesicles (EVs) are powerful biological entities released by cells that contain molecules that can promote changes in their targets. EVs have therefore been studied for clinical applications as vaccines, immunosuppressants, or stimulators of repair and differentiation processes [1–3]. EV is an umbrella term that includes a variety of different released vesicles such as exosomes and microvesicles (MVs). The term “exosomes” is often used to describe vesicles that originate from the fusion of endosomal-originated multivesicular bodies with the plasma membrane. This biogenesis sets them apart from other EVs, for example, those that are released through the budding of the plasma membrane, which are usually referred to as MVs [4]. Because of their distinct biogenesis, MVs are usually larger than exosomes (typically, exosomes are less than 200 nm in diameter, while MVs can range in size up to 1000 nm in diameter), but overlapping of these size ranges can occur. Different isolation protocols focus on the separation of a fraction enriched in exosomes, MVs, or both [5]. Because the study of EVs is a recent field, many studies have used heterogeneous nomenclature when reporting data regarding EVs. It is common to find terms such as “exosomes,” “MVs,” and “microparticles” referring to an indistinct population of EVs [5]. For the sake of uniformity, we are here including all of these studies under the term “EVs.”

Although many functions have been ascribed to EVs, especially involvement in cellular communication, their roles in vivo are still poorly understood. There are likely still major functions and effects that remain unknown, and the immunological effects of EVs released by different cells in pathological states are still poorly studied. On the other hand, because the sorting of molecules to these vesicles and the patterns of EV release are known to be reflective of their originating cell type and physiological state, the EV fraction of extracellular fluids can be very informative. Consequently, substantial attention has been directed towards the use of “liquid biopsies” containing EVs from injured tissue and tumors for detection of disease biomarkers in the hope of developing less invasive diagnostic procedures with high sensitivity and specificity [6]. The sorting of molecules into EVs is still a somewhat obscure process, but it clearly involves the enrichment of distinct proteins and nucleic acids. Particular attention has been given to the protein and RNA content of EVs as agents for altering gene and protein expression in target cells [7].

Currently, the focus on secreted vesicles from stem cells has been most extensively directed to mesenchymal stem cells (MSCs), which are also called mesenchymal stromal cells. These cells are multipotent cells that can be isolated from a variety of adult tissues [8]. The most studied MSCs are isolated from bone marrow (BM-MSCs), adipose tissue (AD-MSCs), or umbilical cord blood (UC-MSCs). The isolated MSCs have been generally heterogeneous and containing stem cells, committed progenitors, and differentiated cells [9]. Hereafter, we will discuss MSCs broadly and independently of their tissue of origin. When not mentioned in the text, the tissue from which the cells were derived is stated in the tables included in this review.

Although there are no specific markers for MSCs, they are usually characterized by their ability to differentiate into at least three lineages of cells of mesodermal origin (osteoblasts, chondroblasts, and adipocytes) upon chemical induction in vitro [10] as well as by the absence of hematopoietic lineage markers but the presence of surface-associated markers such as CD44 and CD90 [11]. MSCs support their niches in vivo by nurturing and promoting the proliferation and differentiation of surrounding cells. When transplanted for cell therapy, these cells migrate to sites of inflammation and injury and are well known for their ability to promote immunomodulation and tissue repair in a wide range of disease models [12]. Nevertheless, they typically do not permanently engraft in the injured tissue when transplanted without a scaffold, and thus they only transiently influence the target tissues.

The secretomes of MSCs and their vesicles are of particular interest because these cells are mostly intended to be used for cell therapy due to their paracrine/endocrine effects rather than their differentiation potential [13–16]. Besides the soluble factors present in these cells’ secretomes, such as growth factors and cytokines, the supernatant of MSC cultures is enriched with EVs. Many examples of pre-clinical data suggest that the EVs derived from MSCs carry over the therapeutic effects of their originating cells, and using EVs instead of the cells themselves can have advantages such as:

- Bypassing most of the safety concerns with regard to cell therapy, such as cellular contamination with oncogenic cells and uncontrolled cell division [16];
- Enabling a wide range of potential manipulations of the particles for improvements in delivery and desired effect; and
- Facilitating the development of methods to optimize the use of MSCs to obtain a higher yield of final therapeutic product because these cells often require invasive procedures in order to be harvested [17].

MSCs are also very responsive to environmental changes, showing different secretion profiles and phenotypes upon different stimuli in vitro, which can be related to their great dynamics in responding to different inflammatory or injured environments in vivo. Treatment of MSCs with EVs derived from other cells such as mast cells and epithelial cells influences their phenotype, as do treatments with soluble factors and changes in the cell culture conditions [18, 19]. It would be of great interest for the scientific community to have more control over MSCs’ immunomodulation and differentiation abilities in order to design more effective and specific treatment strategies, both for direct cell therapy and for EV-mediated therapy.

MSC-derived EVs have emerged as an attractive mediator of immunomodulation and regenerative effects in various animal models. EV-based approaches have already been recognized as a safe and attractive therapeutic intervention, but one significant limitation is the typically low yield of EVs. To overcome this, several high-throughput procedures have been applied for large-scale EV production. Recent studies have utilized EV-mimetic nanovesicles produced from adipose stem cells as well as tumor cells by serial extrusion in order to overcome the low yield normally associated with naturally produced EVs [20–22]. Moreover, in many previous and ongoing studies, a variety of biophysical and biochemical cues have been shown to contribute to the therapeutic effect of EVs and to increase their level of production.

**Fig. 1:**
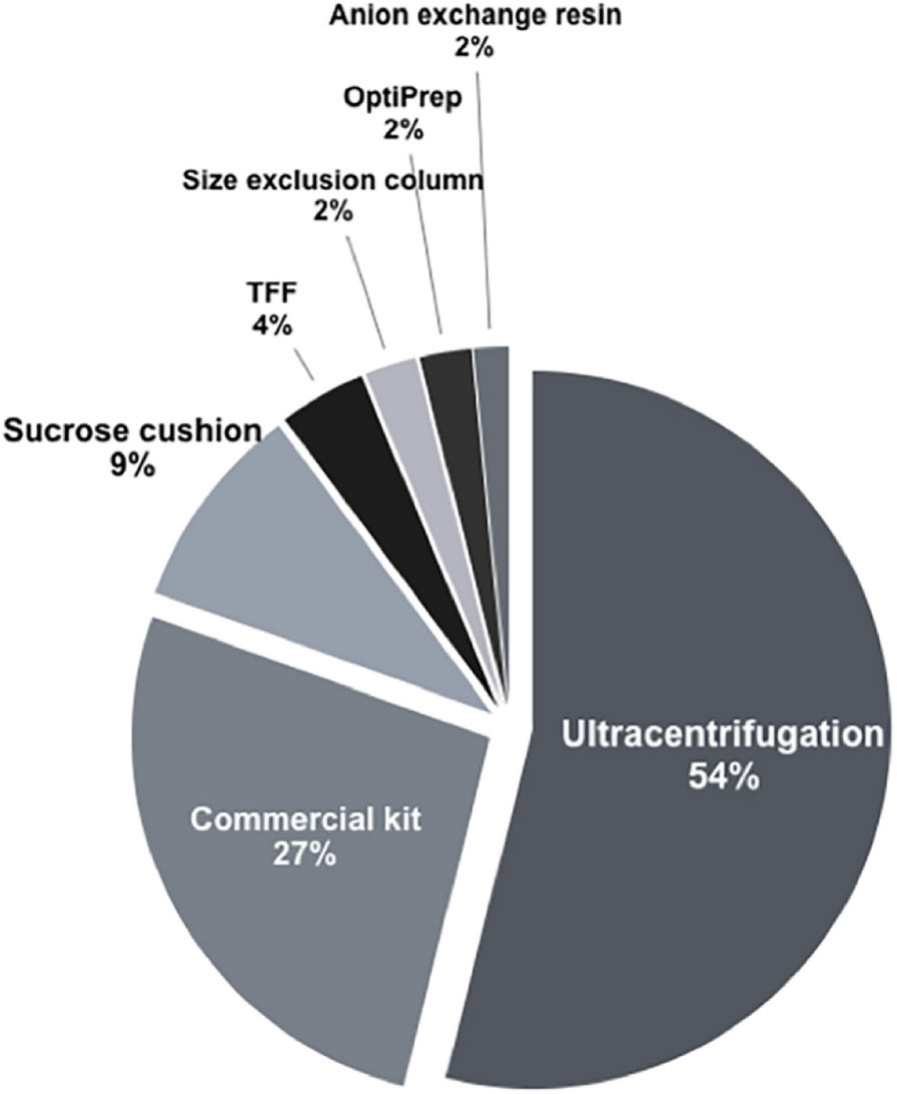
Experimental methods for isolation of MSC-EVs. A total 126 articles were used to analyze the isolation method. Most studies have been conducted using EVs isolated by ultracentrifugation and with commercial kits such as ExoQuick-TC. TFF, tangential flow filtration

EV-based therapy also faces challenges regarding the purity of EV preparations [23]. Our PubMed literature search with the terms “MSCs + extracellular vesicles + exosomes + microvesicles” identified several different methods for EV isolation (Fig. 1). More than half of the articles used only ultracentrifugation, and about 27% used commercial kits, mostly based on protein precipitation protocols. Only about 19% of the articles used some method that separated free secreted proteins from EVs (e.g., density gradient, filtration, and anion-exchange). In order to bring unanimity to our common knowledge of EV-derived functions, it is essential to study the true components of MSC-derived EVs separately from secreted proteins. Producing clean MSC-EV preparations will accelerate the translation of basics findings into clinic practice.

In this review, we discuss the development of MSC-derived extracellular vesicles (MSC-EVs) for therapeutic applications. First, we will discuss components of MSC-EVs and their roles in different in vivo and in vitro models, and then we will discuss some of the possibilities for manipulating MSCs in order to improve or alter their secretion of EVs and thus improve their therapeutic potential.

### Wherein lies the therapeutic potency of MSC-EVs?

**Fig. 2:**
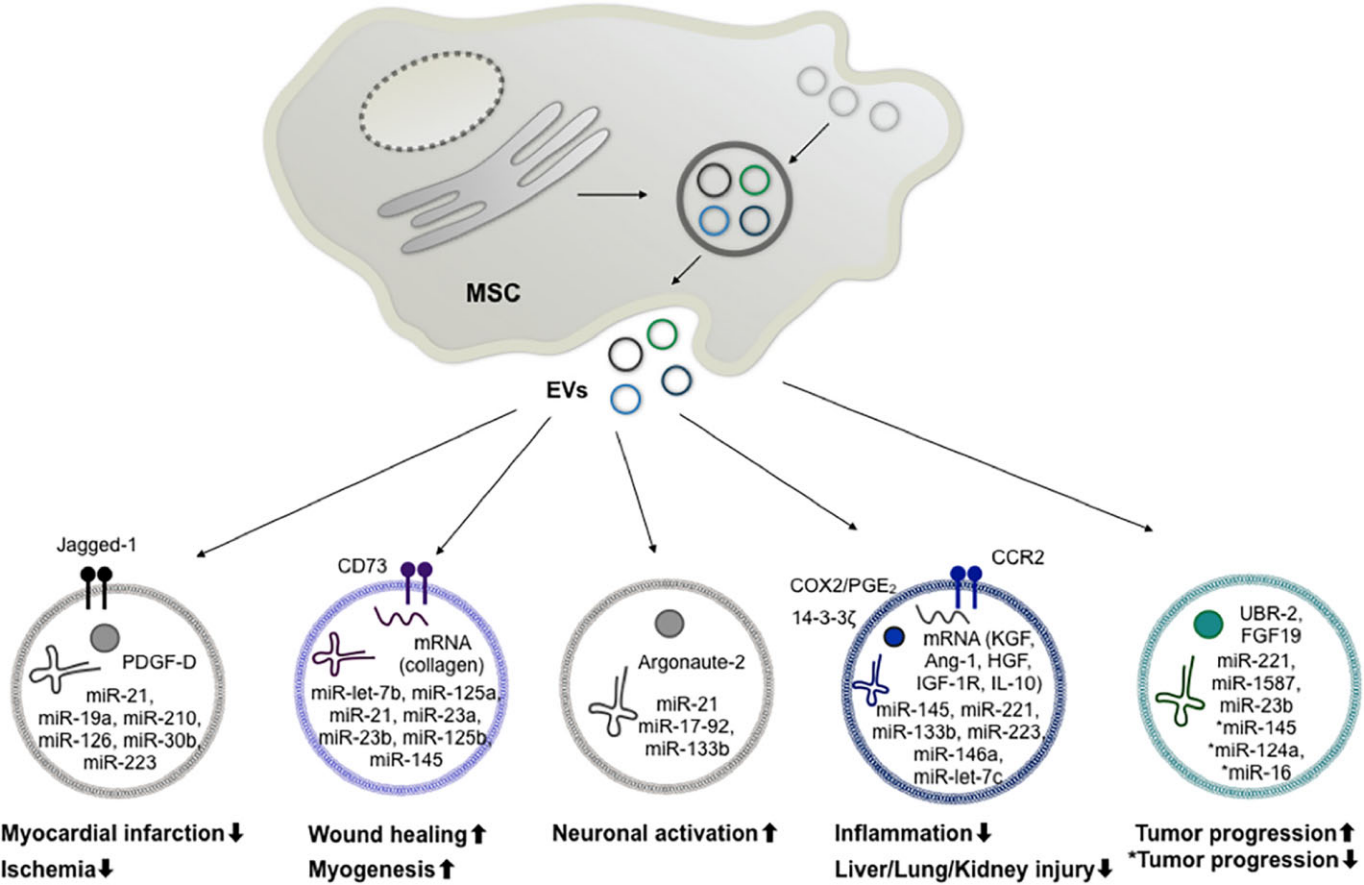
Components of MSC-derived EVs and their related therapeutic potential. The different circles show the suggested vesicular protein, mRNA, and miRNA components mediating the immune modulation, regeneration, and tumor growth effects of MSC-EVs. Abbreviations: MSC, mesenchymal stem cell; EVs, extracellular vesicles; PDGF-D, platelet-derived growth factor-D; COX2, cyclooxygenase 2; PGE_2_, prostaglandin E2; CCR2, C-C chemokine receptor type 2; KGF, keratinocyte growth factor; Ang-1, angiopoietin-1; HGF, hepatocyte growth factor; IGF-1R, insulin-like growth factor 1 receptor; IL-10, interleukin-10; UBR2, ubiquitin protein ligase E3 component n-recognin 2; FGF19, fibroblast growth factor 19

MSCs fulfill their roles in the body via direct cell-to-cell crosstalk as well as through the secretion of an extensive spectrum of soluble factors [24]. Major soluble mediators secreted by MSCs include cytokines, growth factors, and miRNA, which have a wide variety of therapeutic effects ranging from tumor modulation, immunosuppression, and angiogenesis to tissue regeneration [25–27]. Recently, several groups have begun to find another functional component in conditioned media (CM) from MSCs apart from these soluble factors. Bruno et al. showed that fractioned MSC-CM by ultracentrifugation suppressed acute tubular injury in mice, and this pelleted fraction included nanosized vesicular structures [28]. Another group utilized the EV fraction acquired by HPLC-derived size exclusion, which included vesicles with EV marker proteins, to reduce the size of acute myocardial infarction, which had already been accomplished in a previous study using MSCs and soluble factors [29]. In addition to the aforementioned studies using MSC-CM to identify the therapeutically functional EVs, there are about 126 published articles that address the therapeutic function of EVs in a variety of disease models. Here, we will highlight the MSC-EV-associated cargos (proteins and nucleic acids) that have been shown to have distinct functional effects (Fig. 2, Table 1, and Additional file 1: Table S1).

**Table 1:** Overview of MSC-EV-related studies conducted in animal models and in vitro for various diseases

MSC origin	Model	In vivo/in vitro potency	Associated molecule	Ref.
H - AD	Angiogenesis	Increased angiogenic capacity of endothelial cells	miR-125a	[122]
H - AD	Prostate cancer	Decreased proliferation and increased apoptosis	miR-145	[65]
H - BM	Optic nerve crush	Promoted regeneration of retinal ganglion cells axons	Argonaute-2	[34]
H - BM	Leukocyte activation (in vitro)	Decreased inflammatory cytokines in leukocytes	COX2/PGE_2_	[35]
H - BM	Nasopharyngeal carcinoma	Promoted nasopharyngeal carcinoma cell growth	FGF19	[38]
H - BM	Breast cancer	Inhibited endothelial cell migration and tube formation using supernatants from EV-treated breast cancer cells	miR-100	[123]
H - BM	Intervertebral disc degeneration	Inhibited nucleus pulposus cell apoptosis	miR-21	[54]
H - BM	Cardiomyocyte contractility (in vitro)	Increased contractility	miR-21p	[124]
H - BM	Gastric cancer	Increased gastric cancer cell migration and invasion	miR-221	[69]
H - BM	Metastatic breast cancer	Induced dormancy	miR-23b	[125]
H - BM	Acute myeloid leukemia	Different patterns of miRNA expression in EVs	miR-26a-5p, miR-101-3p, miR-23b-5p, miR-339-3p, miR-425-5p	[126]
H - BM	Skeletal muscle regeneration	Increased myogenesis and angiogenesis	miR-494	[127]
H - BM	Acute kidney injury	Recovery from renal injury	mRNA (CCNB1, CDK8, CDC6)	[128]
H - DP	Ischemia	Increased angiogenesis	Jagged1	[31]
H - EMB	Osteochondral defect	Increased cartilage repair	CD73	[33]
H - END	Cardiac infarction (in vitro)	Anti-apoptotic/anti-angiogenic effects and cardioprotection	miR-21	[59]
Human glioma	Glioma stem cell activation (in vitro)	Increased glioma stem cell tumorigenicity	miR-1587	[129]
H - PL	Hindlimb ischemia	Increased proangiogenic effect	VEGF/miR-126	[92]
H - UC	Myocardial infarction	Increased endothelial cell migration and tube formation	PDGF-D	[32]
H - UC	Cisplatin-injured renal tubular epithelial cells (in vitro)	Protected against cisplatin-induced injury in renal tubular epithelial cells	14-3-3ζ	[37]
H - UC	Hypoxia-ischemia (in vitro)	Anti-apoptotic effect	miR-let-7e, miR-let-7a	[130]
H - UC	Hepatitis (in vitro)	Protected against infection by hepatitis C virus	miR-let-7f, miR-145, miR-199a, miR-221	[66]
H - UC	Sepsis	Increased survival in mice and decreased inflammatory cytokines in macrophages	miR-146a	[85]
H - UC	Skin defect	Reduced scar formation and myofibroblast development	miR-21, miR-23a, miR-125b, miR-145	[56]
H - UC	Skin defect in diabetes	Promoted healing of cutaneous wounds	miR-let-7b	[84]
M - BM	Acute kidney injury	Recovery from renal injury	CCR2	[36]
M - BM	Kidney transplantation	Increased graft survival	miR-146a	[131]
M - BM	Systemic sclerosis	Increased osteogenesis and decreased adipogenesis	miR-151-5p	[132]
M - BM	Breast cancer	Decreased angiogenesis	miR-16	[133]
M - BM	Hematopoietic cell activation (in vitro)	Decreased autophagy and rejuvenating effects depending on age	miR-17, miR-34a (negative effect), RNA (positive effect)	[134]
M - BM	Alzheimer’s disease	Prevented cognitive decline	miR-21	[55]
M - BM	Myocardial infarction	Promoted cardiac protection	miR-210	[91]
M - BM	Hindlimb ischemia	Restored blood perfusion and promoted angiogenesis	miR-210-3p, VEGF	[135]
M - BM	Cardiac infarction	Decreased cardiac fibrosis	miR-22	[136]
M - BM	Sepsis	Recovered cardiac function	miR-223	[137]
M - BM	Gastric cancer	Increased proliferation and migration	UBR2	[39]
M - EMB	Angiogenesis	Increased angiogenic capacity of endothelial cells	miR-30b	[138]
R - AD	Erectile dysfunction in diabetes	Restored erectile function	miR-126, miR-130a, miR-132, miR-let7b, miR-let7c	[139]
R - BM	Renal fibrosis (in vitro)	TGF-β induced epithelial mesenchymal transition in HK2 cells	miR-294, miR-133b-3p	[140]
R - BM	Stroke	Neuroprotective effects	miR-133b	[141]
R - BM	Middle cerebral artery occlusion	Promoted neurite outgrowth	miR-133b	[142]
R - BM	Colitis	Decreased colitis-associated fibrosis	miR-200b	[143]
R - BM	Ischemic cardiomyopathy (in vitro)	Reduced oxidative injury	miR-21	[58]

Abbreviations: *MSC* mesenchymal stem cell, *EVs* extracellular vesicles, *H* human, *M* mouse, *R* rat, *BM* bone marrow, *AD* adipose tissue, *DP* dental pulp, *EMB* embryonic, *END* endometrial, *PL* placental, *UC* umbilical cord, *COX2* cyclooxygenase 2, *PGE*_*2*_ prostaglandin E_2_, *FGF19* fibroblast growth factor 19, *CCNB1* cyclin B1, *CDK8* cyclin-dependent kinase 8, *CDC6* cell division cycle 6, *VEGF* vascular endothelial growth factor, *PDGF-D* platelet-derived growth factor-D, *CCR2* C–C chemokine receptor type 2, *UBR2* ubiquitin protein ligase E3 component n-recognin 2, *TGF* tumor growth factor

#### Protein effectors within MSC-EVs

EVs generally include integral membrane proteins such as tetraspanins, peripheral membrane proteins, and cytosolic proteins, and changes in the protein composition of EVs have been shown to be associated with important functional changes [30]. MSC-EVs also harbor numerous protein components that have been suggested to be linked with recovery from many diseases.

Vesicular protein effectors have been explored as a treatment for ischemia and myocardial infarction by promoting angiogenesis. For example, EVs from dental pulp-derived MSCs harbor the Jagged-1 ligand protein, which is an activator of Notch signaling, and they were shown to be effective in activating angiogenic signals [31]. Jagged-1-containing EVs triggered transcriptional changes in Notch target genes in endothelial cells, resulting in induced angiogenesis and capillary-like tube information, and this angiogenic effect could be blocked with an anti-Jagged-1 antibody. In addition to this, UC-MSC-EVs have been shown to carry platelet-derived growth factor-D (PDGF-D), which has been shown to be effective in assisting tissue repair functions in infarcted heart cells [32]. The recovery was abrogated by EVs isolated from MSCs transfected with PDGF-D-siRNA, thus suggesting that PDGF-D/PDGF receptor interactions might play a crucial role in EV-mediated myocardial repair.

In the context of bone regeneration, the therapeutic effect of vesicular CD73 is demonstrated by Zhang et al., in which CD73 present on EVs from embryonic stem cell-derived MSCs was able to repair osteochondral defects in chondrocyte cultures together with greater infiltration of macrophages with an anti-inflammatory phenotype. The role of CD73 in EVs was confirmed by Akt and extracellular signal-related kinase (Erk) signaling using a CD73 inhibitor [33]. Also, a neuronal regeneration study was conducted to investigate the effect of BM-MSC-EVs for treating traumatic and degenerative ocular disease. It was shown that EVs harboring the argonaute-2 (AGO-2) protein promoted significant survival of retinal ganglion cells and regeneration of their axons. The effect was diminished by EVs from MSCs after knockdown of AGO-2, suggesting that AGO-2 is involved in the regenerative effects of EVs [34].

On the basis of MSCs’ well-known immunomodulatory effects, MSC-EVs have also been described as anti-inflammatory agents, thus rationalizing the use of EVs for the treatment of immune diseases, including renal injury. Harting et al. showed that the expression of cyclooxygenase 2 and prostaglandin E2 was increased in BM-MSC-EVs, and these components partially contributed to the attenuation of pro-inflammatory cytokines in splenocytes [35]. Moreover, the quenching effect of the pro-inflammatory cytokine CCL2 by its receptor present on BM-MSC-EVs led to reduced macrophage activation and assisted in the repair of acute renal injury [36]. In addition, delivery of 14-3-3ζ via EVs prevented the autophagic tubule epithelial cell injury that is normally induced by the chemotherapy drug cisplatin [37].

Interestingly, MSC-derived EVs are not limited to only being beneficial in terms of tissue repair and anti-inflammatory effects that can be used therapeutically, and cancer cells can effectively exploit the MSCs’ function for their own growth and immune escape. For example, fibroblast growth factor 19, which is present in BM-MSC-EVs, promotes nasopharyngeal carcinoma cell growth [38]. Similarly, BM-MSC-EVs can deliver ubiquitin protein ligase E3 component n-recognin 2, which has proliferative and migratory effects on gastric cancer cells [39].

Overall, MSCs’ protein cargo can exert functional effects directly by quenching some of the factors that are pro-inflammatory or by enhancing anti-inflammatory factors. Some of the effects are likely to be combinatorial effects together with other cargos, thus dissecting these components one-by-one is a way forward in designing more effective MSC-EVs.

#### Nucleic acids within MSC-EVs

##### a. DNA

Many forms of nucleic acids can be found within EVs, including DNA, mRNA, and miRNA. The existence and localization of DNA in EVs is still controversial, and there are no studies pointing towards the participation of DNA in the therapeutic effects of MSC-EVs. Interestingly, despite descriptions of pro-inflammatory effects of foreign DNA present in vesicles from other origins and uptake of them by MSCs [40, 41], we did not find any reports of inflammation induced by MSC-EV-associated DNA, suggesting that these EVs’ immunosuppressive properties might overcome this possible effect or that there is less harmful DNA associated with MSC-EVs.

##### b. mRNA

Few studies have attributed therapeutic effects to mRNAs when compared to the long list of studies that show at least correlations between specific miRNAs and observed outcomes, as can be seen in Table 1. The stoichiometry of nucleic acids in EVs and the minimal concentration of each miRNA or mRNA needed to promote a robust effect in recipient cells is also still a subject of intense investigation [42].

In acute lung injury models and in pneumonia, the mRNA for keratinocyte growth factor (KGF) has been implicated in the immunomodulation observed with MSC-EV treatment [43, 44]. In these studies, administration of an anti-KGF neutralizing antibody together with the treatment abrogated the beneficial effect initially observed on survival, and pretreatment of MSCs with siRNA against KGF transcripts also partially inhibited the anti-inflammatory effects of MSC-EVs as evidenced by bronchoalveolar lavage fluid cellularity and the presence of inflammatory cytokines. The authors further hypothesized that transcripts for angiopoietin-1, which is also abundant in MSC-EVs, play an important role in restoring lung protein permeability and in resolving inflammation through the use of MSC-EVs in vitro [45] and in a murine model of acute lung injury [46]. In fact, angiopoietin-1 siRNA pretreatment of MSCs or MSC-EVs led to a decrease in immunomodulation and permeability recovery across human lung microvascular endothelial cells in these models.

In an in vitro model of acute kidney injury, Ju and co-workers have suggested a particular role for hepatocyte growth factor mRNA because vesicles treated with RNase were shown to be ineffective in promoting dedifferentiation and subsequent growth of tubular cells [47]. In another in vitro model of acute kidney injury induced by cisplatin, it was found that EV-associated mRNA for insulin-like growth factor 1 receptor was important for the protection of proximal tubular epithelial cells [48]. In a similar cisplatin-induced in vitro model, interleukin (IL)-10 mRNA was also found to be transferred through MSC-EVs [49].

Furthermore, mRNA for the synthesis of type VII collagen was found to be transferred in vitro to recessive dystrophic epidermolysis bullosa cells together with the collagen protein itself [50]. This condition is characterized by loss-of-function mutations in the type VII collagen gene, and MSC-EVs might therefore be a potential treatment for this disease.

##### c. miRNA

Increasing evidence has been provided for the effectiveness of miRNAs contained within MSC-EVs. Many miRNAs that are involved in the therapeutic effects of MSC-EVs in different disease conditions are shown in Fig. 2 and Table 1.

Because the field of miRNA has been most extensively explored in cancer-related research, some of these miRNAs are known to be upregulated or are suggested to be markers in specific cancer types. However, this does not necessarily mean that the presence of these miRNAs in EVs represent a pro-tumorigenic risk because it is often the combination of multiple factors that is important for defining the ultimate role of each molecule in this process. Nevertheless, it is important to keep in mind that oncogenic molecules might be transferred through EVs and might influence the development of tumors when there is a lack of onco-suppressor genes in vivo [51]. On the other hand, there might be only transient effects of this transfer in non-mutated cells [52].

Generally speaking, among the miRNAs that are most frequently associated with the therapeutic properties of MSC-EVs, miR-21, miR-19a, and miR-210 are linked to cardiovascular diseases; miR-let-7b, miR-125a, and miR-21 are linked to wound healing; miR-21, miR-17-92, and miR-133b are linked to neural damage; miR-223, miR-146a, and miR-let-7c are linked to protection against hepatic and renal injuries; and miR-221, miR-1587, and miR-23b are linked to cancer-related effects (Fig. 2). Here, we will discuss in depth some of the miRNAs that are most often cited as possible mediators of MSC-EVs’ effects.

- miR**-**21

Given that miR-21 has been shown to regulate cell survival by stimulating proliferation and by inhibiting apoptosis in different cell types [53], the contribution of this miRNA has been connected with MSC-EV-mediated therapeutic effects in various disease models. BM-MSCs have been shown to deliver exogenous miR-21 via EVs and thus to prevent nucleus pulposus cell apoptosis and to reduce intervertebral disc degeneration [54]. In addition, the expression of miR-21 has been shown to increase in MSC-EVs under hypoxic conditions, and injection of these MSC-EVs could reduce cognition and memory impairment in mice together with reduced plaque deposition and reduced activation of microglia [55].

The function of miR-21 was further described by Fang et al. and Jackson et al. that EVs from UC-MSCs enriched with miR-21 play a key role in suppressing myofibroblast formation and thus in preventing excessive scar formation [56, 57]. Blocking miR-21 in these EVs abolished the ability of EVs to inhibit myofibroblast formation, suggesting that this specific miRNA is essential for the anti-scarring functions of MSCs.

miR-21 has also been described as having a protective role in cardiac injuries. EVs derived from BM-MSCs harbored increased levels of miR-21 after hydrogen peroxide-induced oxidation, and vesicular miR-21 could be transported to cardiac stem cells in order to functionally inhibit phosphatase and tensin homolog (PTEN) expression and thus protect against oxidative stress-triggered cell death [58]. Another study showed that selective antagonism of miR-21 by anti-miR treatment eliminated the anti-apoptotic and angiogenic effects of MSC-EVs with subsequent upregulation of PTEN, a miR-21 target, suggesting that miR-21 might be a potential mediator of MSC-EVs’ therapeutic effects against cardiovascular diseases [59].

- miR**-**145

miR-145 is related to the processes of cellular differentiation and the activation of smooth muscle cells and myofibroblasts [60, 61]. Moreover, miR-145 is often described as having tumor suppression effects [62–64]. In agreement with these finding, upregulation of miR-145 in MSC-EVs has been shown to be effective in skin defect healing and to have anti-tumor effects in prostate cancer [56, 65]. miR-145 is enriched in UC-MSC-derived EVs as determined by high-throughput RNA sequencing [56]. Overexpression of miR-145 in EVs could suppress the activation of tumor growth factor (TGF)-β/SMAD2 leading to the inhibition of differentiation of fibroblasts into myofibroblasts, and depletion of this miRNA greatly abolished the ability of EVs to inhibit the TGF-β/SMAD2 pathway.

In terms of cancer prevention, AD-MSC-derived EVs significantly inhibited the proliferation of metastatic prostate cancer through apoptosis, and this effect was negated by miR-145 knockdown leading to reduced expression of Caspase 3/7 and increased expression of anti-apoptotic proteins [65]. Interestingly, EVs secreted from UC-MSCs have been shown to inhibit hepatitis C virus (HCV) infection by suppressing viral infection, and this was largely attributed to suppression of viral RNA replication by miR-145 [66].

- miR**-**221

In contrast to miR-145, the facilitating role of miR-221 in cancer progression has been extensively recognized in recent years. For example, CD44 expression in hepatocellular carcinoma is controlled by miR-221 through the PI3K-Akt-mTOR pathway [67]. Additionally, miR-221 can support non-small-cell lung carcinoma by targeting tissue inhibitor of metallopeptidases-2 [68]. Similarly, high expression of miR-221 in EVs from BM-MSCs has been shown to effectively increase gastric cancer cell migration, invasion, and adhesion to the extracellular matrix [69]. Another study using miR-221 showed that upregulated miR-221 in MSC-EVs protected against HCV in a similar manner as miR-145 mentioned above [66].

### How to make MSC-based therapies more potent?

#### Biophysical cues

**Fig. 3:**
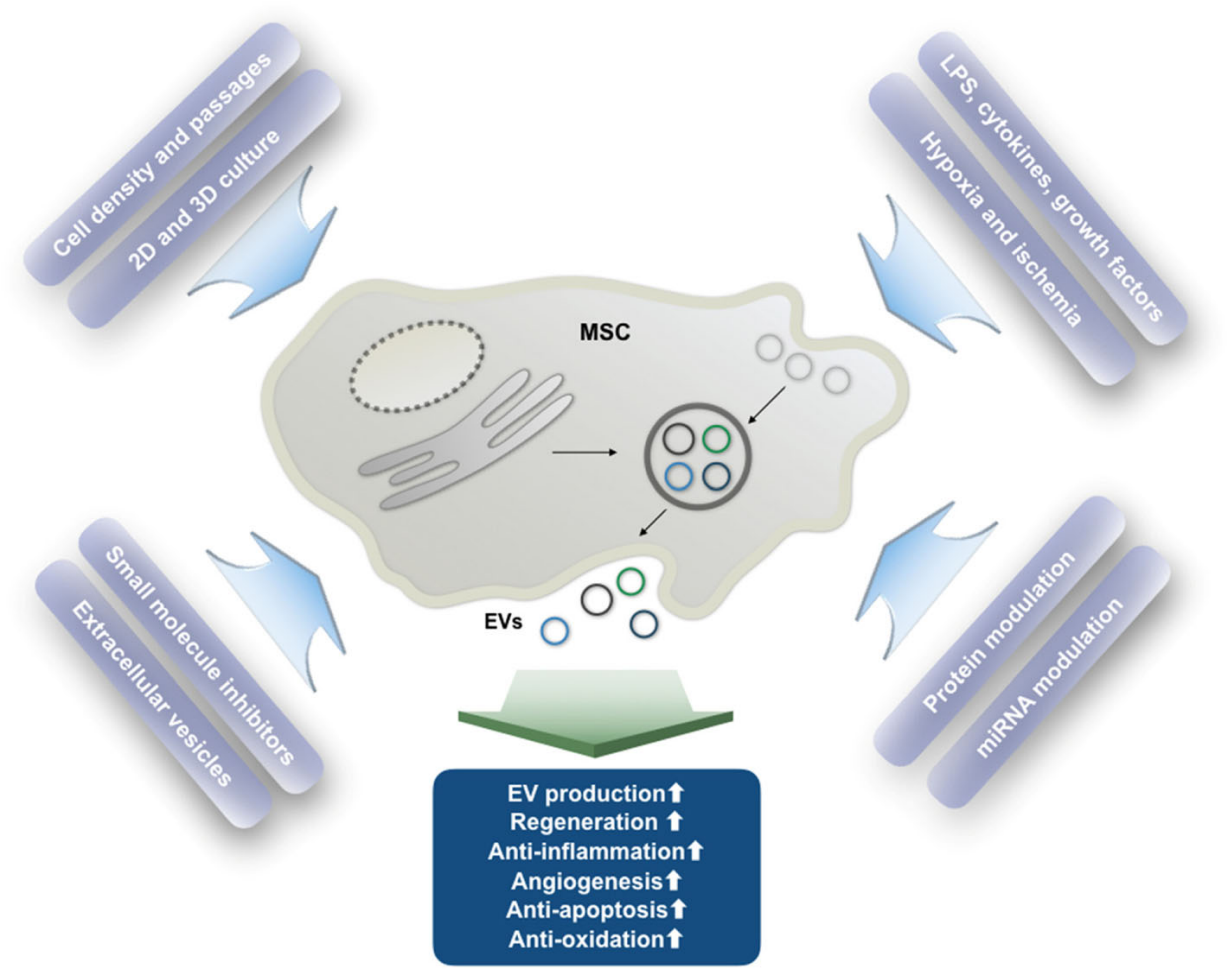
The influence of various conditions on the production and function of EVs. The effects of culture conditions and external stimuli on MSC-EV secretion profiles and functional changes. MSC, mesenchymal stem cell; EVs, extracellular vesicles; LPS, lipopolysaccharide

MSCs have been shown to be stimulated by a variety of different biophysical and biochemical stimuli (Fig. 3). Biophysical inducers include electric pulsing [70, 71], low-power laser irradiation [72], non-coherent red light [73], electromagnetic field exposure [74], mechanical cues (e.g., fluidics, tension, and pressure) and substrate topography and stiffness [75], 2D and 3D scaffolds/scaffold-free culture [76, 77], and magnetic forces [78]. Upon these different treatments, MSCs might dramatically change their phenotype and begin to differentiate into specific types of cells, which is useful for a range of applications such as tissue regeneration, especially in injuries to organs with mesenchymal origins [79, 80]. Nevertheless, some of these changes in the biophysical parameters of MSC culture can also influence their secretion profiles without promoting complete differentiation. Many of these treatments can, for example, increase the proliferation of MSCs, but little is known about their effects on EV secretion or their immunomodulation abilities, leaving a wide range of conditions to be explored in attempts to increase MSC-EV yields and to control their contents.

In a study of EVs derived from MSCs subjected to 3D culture in type I collagen scaffolds versus common 2D cultures, the authors isolated EVs from their supernatants using a commercial kit and found greater amounts of protein and better outcomes in promoting functional recovery and immunomodulation in a model of traumatic brain injury in the samples isolated from 3D cultured cells [79].

Another MSC culture parameter that can influence the yield of EVs is cell seeding density, with lower density being related to higher yields. It is, however, still unclear if these effects are related to cell-to-cell contact because multiple culture medium collections instead of one single collection over the same period of time also increases the number of EVs that can be collected. It is possible that EVs or metabolites present in the cell culture biochemically decrease the production and or secretion of EVs by MSCs [80].

#### Biochemical cues

It is thought that MSCs responding to bacteria-derived molecules like lipopolysaccharides and the cytokines released in response to such molecules can increase their therapeutic effect against inflammatory environments [81–83]. More recently, EVs produced from MSCs under inflammatory conditions have gained increasing importance. A study done by Ti et al. has shown that lipopolysaccharide stimulation increases the secretion of EVs from UC-MSCs and enhances M2 macrophage polarization and diabetic cutaneous wound healing [84]. Increasing evidence indicates that inflammatory cytokines might enhance the therapeutic efficacy of MSC-EVs [35, 85, 86]. EVs from IL-1β-pretreated UC-MSCs were shown to have greater immunomodulatory effects than EVs from non-treated MSCs, suggesting that more functional molecules such as miR-146a were embedded in the EVs from IL-1β-pretreated MSCs [85]. In line with this, MSC-EVs cultured in the presence of tumor necrosis factor alpha, interferon gamma, or TGF-β led to significantly decreased cytokine expression in splenocytes and to strongly increased regulatory T cell differentiation that in turn exerted an anti-inflammatory effect [35, 86].

MSC-CM includes various growth factors such as vascular endothelial growth factor and PDGF, and it mimics the beneficial effects associated with intact cells [87, 88]. The increased therapeutic effect induced by pre-stimulation with PDGF has been confirmed by Lopatina et al. on the basis of their work that use the angiogenic potential of AD-MSC-EVs for regenerative medicine [89]. Also, hormone stimulation with erythropoietin increased the production of EVs and enhanced the protective effects of EVs following renal injury compared to untreated EVs [90]. In addition, hypoxic and ischemic conditions have been shown to alter the characteristics of MSCs with respect to EV function. It is reported that hypoxia preconditioning causes BM-MSCs to increase the production of EVs and that these EVs have superior activity in cardiac protection by stimulating neovascularization [91]. Also, EVs released by MSCs during nitric oxide stimulation have been shown to augment the angiogenic effects of endothelial cells and to restore limb function in hindlimb ischemia [92]. Moreover, incorporation of paclitaxel into BM-MSC-EVs was shown to inhibit tumor growth in vitro [93]. In addition, the serum contents of culture media were found to alter the MSC characteristics and the RNA contents of released EVs, suggesting that MSC-EVs can be modulated to contain different active components for future therapeutic applications [94].

EVs derived from differentiated cells are able to modify the characteristics of MSCs. EVs from neuronal cells can mediate MSC neuronal induction via miR-125b transfer [95], and endothelial cell-derived EVs influence MSC proliferation and migration, providing evidence for EVs as a communication channel between endothelial cells and MSCs [96]. In addition, mast cell-derived EVs modulated MSC function to induce anti-inflammatory effects during ovalbumin-induced allergy model via vesicle-associated TGF-β [97]. Moreover, EVs derived from tumor cells can also modulate the MSC phenotype. For example, EVs from cancer stem cells induce increased chemoattraction in MSCs resulting in tumor progression, and EVs from lung cancer cells stimulate the production and secretion of IL-6, IL-8, and monocyte chemoattractant protein-1 in MSCs, thus imbuing MSCs with more tumor-supportive characteristics [98]. However, there are no well-defined studies on how other types of EVs might affect MSC function in terms of MSC-EVs despite the high probability that these EVs from differentiated cells will be able to modulate further EV production by MSCs. Therefore, research into the role of other cell-derived EVs on the potency of MSCs in terms of EV secretion will be needed in order to obtain optimal therapeutic outcomes.

#### Cellular reprogramming of MSCs

Despite the strong therapeutic effects of MSC-EVs, there is a need to further understand how genetic modification of MSCs can increase the therapeutic potency of secreted EVs. Researchers are currently trying to develop more therapeutically optimized MSCs through overexpression of proteins and miRNAs. Here, we will focus on how genetically modified MSC-EVs show altered cargo and improved functional effects.

##### a. Overexpressed proteins in MSCs

Most proteins targeted for overexpression in MSCs have been transcription factors and signaling molecules. MSCs generally have limited expansion capability, thus Lai et al. have created immortalized MSCs by inducing overexpression of c-Myc. The production of EVs from these immortalized cells is scalable under stringent GMP conditions, and this enables these EVs to be used in the clinic [99]. Another study showed that overexpression of the GATA-4 transcription factor in BM-MSCs increased the ability of their secreted EVs to improve cardiac function [100]. Such EVs could transfer more miR-19a than EVs from control MSCs, thus resulting in restored cardiac contractile function and reduced infarct size in a mouse model. In addition, the hypoxia-inducible factor 1-α (HIF-1α) transcription factor is usually stabilized during ischemia and upregulates a variety of cardioprotective genes, and this led researchers to mutate the HIF-1α gene (oxygen-resistant form) in dental pulp-derived MSCs for application in treating ischemia-related disease [101]. EVs from HIF-1α overexpressing MSCs had increased EV marker proteins such as tetraspanins and increased angiogenic activity compared to control EVs, and this led to increased repair of cardiac tissue in a mouse model [31]. A similar study showed that EVs derived from BM-MSCs overexpressing HIF-1α were able to promote bone regeneration and to reduce steroid-induced avascular necrosis of the femoral head [102]. Signaling molecules such as Akt have been exploited in MSCs to increase their effectiveness. EVs from UC-MSCs that overexpress Akt harbor higher levels of Akt than control EVs, and this leads to accelerating proliferation, migration, and vessel formation in endothelial cells thus resulting in greater efficiency of cardiac repair. This effect is mediated by enhanced PDGF-D production in endothelial cells that promotes angiogenesis in the ischemic heart [32]. Some indications of these cardioprotective effects of Akt-overexpressing MSC-EVs were seen in the CM, and the effects were attributed to secreted frizzled-related protein 2 [103].

MSCs reside in close proximity to tumor cells and are reported to be involved in tumor progression [104]. It has been generally considered that the tumor microenvironment can alter the contents of MSC-EVs and lead them towards a more pro-tumorigenic phenotype. For instance, Roccaro et al. showed that BM-MSC-EVs from multiple myeloma patients have different contents of tumor suppressor miRNAs than EVs from normal healthy subjects, and these patient-derived EVs promoted multiple myeloma tumor growth, whereas EVs from healthy individuals inhibited the growth of tumor cells [105]. Thus, genetic modification of MSCs has also been investigated in terms of how they affect tumor growth. Tumor necrosis factor-related apoptosis-inducing ligand (TRAIL) has been shown to be a promising agent for cancer therapy [106], and based on this, Tuan et al. transfected this gene into MSCs and then measured the cancer cell-killing efficacy of EVs derived from these cells. Such EVs were decorated with highly expressed TRAIL and induced apoptosis in various cancer cell lines but not in primary bronchial cells [106, 107]. In addition to gene overexpression, the effect of tumor-related gene knockdown has also been characterized in BM-MSCs. EVs from p53-deficient BM-MSCs were enriched in a UBR2 protein that promotes gastric cancer progression. Such regulation of the p53 oncogene that indirectly targets UBR2 to target cells enhanced tumor growth and metastasis by regulating the Wnt/β-catenin pathway [39].

##### b. Overexpression of miRNA in MSCs

The use of miRNAs that target transcriptional and posttranscriptional regulation might offer a novel option for treating many diseases. However, the advancement of miRNA therapy has been hindered by obstacles in delivering miRNA to the target organs. EVs have emerged as an effective vehicle for delivering miRNA, thus many researchers have been engineering MSCs to load miRNA into EVs and have seen potent therapeutic effects (Table 2).

**Table 2:** Overview of gene-transfected MSC studies conducted in in vitro and in vivo models

MSC origin	Model	In vivo*/*in vitro potency	Transgene	Ref.
H - BM	Glioblastoma	Increased survival in glioma stem cell-injected mice	miR-124a	[144]
H - BM	Breast cancer	Decreased tumor activity and size	miR-379	[145]
H - BM	Renal fibrosis	Decreased matrix deposition	miR-let-7c	[108]
H - SYN	Diabetes skin defect	Increased proliferation of fibroblasts and epithelial cells	miR-126	[117]
H - SYN	Osteoarthritis	Increased cartilage tissue regeneration	miR-140-5p	[116]
H - UC	Glioblastoma (in vitro)	Decreased proliferation and migration and increased chemosensitivity	miR-124	[111]
H - UC	Burn-induced inflammation	Decreased inflammation	miR-181c	[146]
H/M - AD	Liver fibrosis	Inhibited fibrosis	miR-122	[109]
H/M - AD	Hepatocarcinoma	Inhibited tumor growth	miR-122	[110]
Marrow stromal cells	Glioma	Inhibited tumor growth	miR-146b	[112]
M - AD	Liver fibrosis	Increased autophagy	miR-181-5p	[147]
M - BM	Autoimmune hepatitis	Recovery from liver injury	miR-223	[115]
R - BM	Myocardial infarction	Improved cardiac function and reduced infarction size	miR-19a	[100]
R - BM	Cortical neuron activation (in vitro)	Increased axonal growth	miR-17-92	[113]
R - BM	Intracerebral hemorrhage	Neuroprotective effects	miR-133b	[114]
R - BM	Acute myocardial infarction	Increased cardiac function	miR-133	[148]
R - BM	Cardiomyocyte activation (in vitro)	Increased survival after hypoxia in cardiomyocytes	miR-221	[149]

Abbreviations: *MSC* mesenchymal stem cell, *H* human, *M* mouse, *R* rat, *BM* bone marrow, *AD* adipose tissue, *UC* umbilical cord, *SYN* synovial

A mouse renal injury and liver fibrosis model was used to study the anti-fibrotic effect of EV-mediated miR-let7c and miR-122. In this mouse model, EVs released from MSCs, which had been engineered to overexpress miR-let7c, included abundant miR-let7c and were able to attenuate kidney injury and to significantly downregulate the expression of TGF-β1 and downstream fibrotic genes in the kidney, thus providing a prime example of the use of engineered MSCs for therapeutic delivery of miRNA via EVs [108]. Given that miR-122 plays a crucial role in liver fibrosis by negatively regulating the proliferation of hepatic cells, miR-122 was modified in AD-MSCs to produce EVs with increased levels of miR-122. These EVs mediated the communication between MSCs and hepatic stellate cells through miR-122-induced downregulation of target genes such as insulin-like growth factor receptor-1, cyclin G-1, and prolyl-4-hydroxylase α-1 [109].

In addition to the anti-fibrotic effect of EV-associated miR-122, the same miR-122-containing EVs made hepatocellular carcinoma cancer cells more sensitive to the chemotherapeutic effects of sorafenib [110]. In line with this, other miRNA modifications have been shown to endow MSC-EVs with anti-tumor effects. In order to mitigate the difficulties in targeting miRNAs to glioblastoma multiforme, Wharton’s jelly-MSCs were overexpressed with miR-124, and the derived EVs enhanced chemosensitivity to temozolomide and decreased the migration of glioblastoma cells [111]. In another study, rat brain MSC-EVs overexpressing miR-146b were used to reduce the tumor burden of glioma xenografts, and intra-tumor administration of these EVs reduced glioma growth in the rat brain [112].

The neuroprotective activities of miR-17-92 and miR-133 have been augmented in EVs from miRNA-expressing MSCs. EVs harvested from MSCs transfected with miR-17-92 showed significantly increased axonal growth of cortical neurons characterized by higher axonal elongation speed compared to control EVs [113]. In an intracerebral hemorrhage rat model, miR-133-containing MSC-EVs were able to generate a pro-survival signaling response that helped to stop the degeneration of neurons, and this was mediated by suppression of RhoA and activation of the Erk172/cAMP response element-binding protein [114].

Studies have also examined the regenerative effects of miRNAs delivered by MSC-EVs in different disease models. EVs from miR-223-overexpressing BM-MSCs were used in a mouse model of autoimmune hepatitis, and the EVs could prevent liver injury through miR-223-induced downregulation of target cytokine expression and downregulation of NLR pyrin domain containing 3 and caspase-1 activity [115]. Moreover, a study on EVs derived from miR-140-5p-overexpressing human synovial MSCs showed enhanced cartilage tissue regeneration and reduced osteoarthritis of the knee in a rat model [116], and EVs derived from miR-126-overexpressing human synovial MSCs healed full-thickness skin defects in a diabetic rat model [117].

## Conclusions and perspectives

The relationships between EV components and EVs’ biological effects have also been investigated, and the most commonly identified molecules are proteins and miRNAs. Various strategies for exogenously loading isolated EVs with specific proteins and nucleic acids have been investigated [118], for example, electroporation, freeze-thaw cycles, saponin-mediated loading, and hypotonic dialysis [119]. Moreover, many groups have started to pack EVs with desired cargos using transgenic MSCs that are genetically modified to overexpresses certain proteins and miRNAs. However, this requires optimized conditions in order for genetically modified EVs to acquire more effective functional properties. In addition, comprehensive studies are needed regarding the quality control of EV compositions as well as the safety and efficacy of these EVs before they can be used in clinical applications.

Before addressing the benefits of MSC-EVs over MSCs, it is essential to consider the need for careful investigation of the following issue. The effects of various external factors on the properties of MSCs have been described in numerous clinical trials [120]. Subtle differences in donor variance, senescence, cell culture methods, and immunogenicity were shown to make the functional alteration in MSC therapy. For instance, MSCs undergoing cellular senescence promoted metabolic dysfunction [121] and lost their mesenchymal plasticity and anti-inflammatory effect [120], which might be leading to failures of MSC therapy. To our knowledge, there have been no studies assessing the relationship between EV therapeutic activities and MSC senescence. However, it needs further investigation on senescent cell contents when EVs are functionally evaluated, and in-depth understanding of the related mechanism will contribute to successful development of MSC-EVs for clinical use.

In conclusion, MSCs have potential therapeutic functions through various vesicular components together with the cells themselves and their secreted soluble factors, and MSCs are amenable to modifications that improve the quantity and effectiveness of the EVs they produce. Thus MSC-derived EVs can be harnessed as powerful therapeutic agents to deliver anti-inflammatory and regenerative compounds in many different diseases. Future work will focus on developing bioengineered MSCs that produce significantly increased yields of EVs that can safely transfer a wide variety of potent and effective therapeutic molecules.

## Additional file

Additional file 1: Table S1 Overview of MSC-EVs studies. There are total 126 published articles that address the therapeutic function of EVs in a variety of disease models. (XLSX 19 kb)

## Data Availability

Data sharing is not applicable to this article as no datasets were generated or analyzed during the current study.

## Abbreviations

AD: Adipose tissue
AGO-2: Argonaute-2
BM: Bone marrow
CM: Conditioned media
Erk: Extracellular signal related kinase
EVs: Extracellular vesicles
HCV: Hepatitis C virus
HIF: Hypoxia-inducible factor
IL: Interleukin
KGF: Keratinocyte growth factor
MSCs: Mesenchymal stem cells
MVs: Microvesicles
PDGF: Platelet-derived growth factor
PTEN: Phosphatase and tensin homolog
TGF: Tumor growth factor
TRAIL: Tumor necrosis factor-related apoptosis-inducing ligand
UC: Umbilical cord blood

## References

[1] Lener Thomas, Gimona Mario, Aigner Ludwig, Börger Verena, Buzas Edit, Camussi Giovanni, Chaput Nathalie, Chatterjee Devasis, Court Felipe A., Portillo Hernando A. del, O'Driscoll Lorraine, Fais Stefano, Falcon-Perez Juan M., Felderhoff-Mueser Ursula, Fraile Lorenzo, Gho Yong Song, Görgens André, Gupta Ramesh C., Hendrix An, Hermann Dirk M., Hill Andrew F., Hochberg Fred, Horn Peter A., Kleijn Dominique de, Kordelas Lambros, Kramer Boris W., Krämer-Albers Eva-Maria, Laner-Plamberger Sandra, Laitinen Saara, Leonardi Tommaso, Lorenowicz Magdalena J., Lim Sai Kiang, Lötvall Jan, Maguire Casey A., Marcilla Antonio, Nazarenko Irina, Ochiya Takahiro, Patel Tushar, Pedersen Shona, Pocsfalvi Gabriella, Pluchino Stefano, Quesenberry Peter, Reischl Ilona G., Rivera Francisco J., Sanzenbacher Ralf, Schallmoser Katharina, Slaper-Cortenbach Ineke, Strunk Dirk, Tonn Torsten, Vader Pieter, Balkom Bas W. M. van, Wauben Marca, Andaloussi Samir El, Théry Clotilde, Rohde Eva, Giebel Bernd (2015). Applying extracellular vesicles based therapeutics in clinical trials – an ISEV position paper. Journal of Extracellular Vesicles.

[2] Gimona Mario, Pachler Karin, Laner-Plamberger Sandra, Schallmoser Katharina, Rohde Eva (2017). Manufacturing of Human Extracellular Vesicle-Based Therapeutics for Clinical Use. International Journal of Molecular Sciences.

[3] Reiner AT, Witwer KW, van Balkom BWM, de Beer J, Brodie C, Corteling RL (2017). Concise review: developing best-practice models for the therapeutic use of extracellular vesicles. Stem Cells Transl Med.

[4] Lasser C, Jang SC, Lotvall J (2018). Subpopulations of extracellular vesicles and their therapeutic potential. Mol Asp Med.

[5] Crescitelli Rossella, Lässer Cecilia, Szabó Tamas G., Kittel Agnes, Eldh Maria, Dianzani Irma, Buzás Edit I., Lötvall Jan (2013). Distinct RNA profiles in subpopulations of extracellular vesicles: apoptotic bodies, microvesicles and exosomes. Journal of Extracellular Vesicles.

[6] Garcia-Romero N, Esteban-Rubio S, Rackov G, Carrion-Navarro J, Belda-Iniesta C, Ayuso-Sacido A (2018). Extracellular vesicles compartment in liquid biopsies: clinical application. Mol Asp Med.

[7] Riazifar M, Pone EJ, Lotvall J, Zhao W (2017). Stem cell extracellular vesicles: extended messages of regeneration. Annu Rev Pharmacol Toxicol.

[8] da Silva ML, Chagastelles PC, Nardi NB (2006). Mesenchymal stem cells reside in virtually all post-natal organs and tissues. J Cell Sci.

[9] Squillaro T, Peluso G, Galderisi U (2016). Clinical trials with mesenchymal stem cells: an update. Cell Transplant.

[10] Robey P. "Mesenchymal stem cells": fact or fiction, and implications in their therapeutic use. F1000Res. 2017;6. 10.12688/f1000research.10955.1 PMC5399967 28491279

[11] Dominici M, Le Blanc K, Mueller I, Slaper-Cortenbach I, Marini F, Krause D (2006). Minimal criteria for defining multipotent mesenchymal stromal cells. The International Society for Cellular Therapy position statement. Cytotherapy..

[12] Prockop DJ (2009). Repair of tissues by adult stem/progenitor cells (MSCs): controversies, myths, and changing paradigms. Mol Ther.

[13] Camussi G, Deregibus MC, Tetta C (2010). Paracrine/endocrine mechanism of stem cells on kidney repair: role of microvesicle-mediated transfer of genetic information. Curr Opin Nephrol Hypertens.

[14] Meirelles Lda S, Fontes AM, Covas DT, Caplan AI (2009). Mechanisms involved in the therapeutic properties of mesenchymal stem cells. Cytokine Growth Factor Rev.

[15] Timmers L, Lim SK, Hoefer IE, Arslan F, Lai RC, van Oorschot AA (2011). Human mesenchymal stem cell-conditioned medium improves cardiac function following myocardial infarction. Stem Cell Res.

[16] Togel F, Weiss K, Yang Y, Hu Z, Zhang P, Westenfelder C (2007). Vasculotropic, paracrine actions of infused mesenchymal stem cells are important to the recovery from acute kidney injury. Am J Physiol Renal Physiol.

[17] Yeo RW, Lai RC, Zhang B, Tan SS, Yin Y, Teh BJ (2013). Mesenchymal stem cell: an efficient mass producer of exosomes for drug delivery. Adv Drug Deliv Rev.

[18] Chiabotto G, Bruno S, Collino F, Camussi G (2016). Mesenchymal stromal cells epithelial transition induced by renal tubular cells-derived extracellular vesicles. PLoS One.

[19] Hu C, Li L (2018). Preconditioning influences mesenchymal stem cell properties in vitro and in vivo. J Cell Mol Med.

[20] Jang SC, Kim OY, Yoon CM, Choi DS, Roh TY, Park J (2013). Bioinspired exosome-mimetic nanovesicles for targeted delivery of chemotherapeutics to malignant tumors. ACS Nano.

[21] Lunavat TR, Jang SC, Nilsson L, Park HT, Repiska G, Lasser C (2016). RNAi delivery by exosome-mimetic nanovesicles - implications for targeting c-Myc in cancer. Biomaterials..

[22] Kim YS, Kim JY, Cho R, Shin DM, Lee SW, Oh YM (2017). Adipose stem cell-derived nanovesicles inhibit emphysema primarily via an FGF2-dependent pathway. Exp Mol Med.

[23] Lobb RJ, Becker M, Wen SW, Wong CS, Wiegmans AP, Leimgruber A (2015). Optimized exosome isolation protocol for cell culture supernatant and human plasma. J Extracell Vesicles..

[24] Smirnov SV, Harbacheuski R, Lewis-Antes A, Zhu H, Rameshwar P, Kotenko SV (2007). Bone-marrow-derived mesenchymal stem cells as a target for cytomegalovirus infection: implications for hematopoiesis, self-renewal and differentiation potential. Virology..

[25] Rhee KJ, Lee JI, Eom YW (2015). Mesenchymal stem cell-mediated effects of tumor support or suppression. Int J Mol Sci.

[26] Ghannam S, Bouffi C, Djouad F, Jorgensen C, Noel D (2010). Immunosuppression by mesenchymal stem cells: mechanisms and clinical applications. Stem Cell Res Ther.

[27] Tao H, Han Z, Han ZC, Li Z (2016). Proangiogenic features of mesenchymal stem cells and their therapeutic applications. Stem Cells Int.

[28] Bruno S, Grange C, Deregibus MC, Calogero RA, Saviozzi S, Collino F (2009). Mesenchymal stem cell-derived microvesicles protect against acute tubular injury. J Am Soc Nephrol.

[29] Lai RC, Arslan F, Lee MM, Sze NS, Choo A, Chen TS (2010). Exosome secreted by MSC reduces myocardial ischemia/reperfusion injury. Stem Cell Res.

[30] Bissig C, Gruenberg J (2013). Lipid sorting and multivesicular endosome biogenesis. Cold Spring Harb Perspect Biol.

[31] Gonzalez-King H, Garcia NA, Ontoria-Oviedo I, Ciria M, Montero JA, Sepulveda P (2017). Hypoxia inducible factor-1alpha potentiates jagged 1-mediated angiogenesis by mesenchymal stem cell-derived exosomes. Stem Cells.

[32] Ma J, Zhao Y, Sun L, Sun X, Zhao X, Sun X (2017). Exosomes derived from Akt-modified human umbilical cord mesenchymal stem cells improve cardiac regeneration and promote angiogenesis via activating platelet-derived growth factor D. Stem Cells Transl Med.

[33] Zhang S, Chuah SJ, Lai RC, Hui JHP, Lim SK, Toh WS (2018). MSC exosomes mediate cartilage repair by enhancing proliferation, attenuating apoptosis and modulating immune reactivity. Biomaterials..

[34] Mead B, Tomarev S (2017). Bone marrow-derived mesenchymal stem cells-derived exosomes promote survival of retinal ganglion cells through miRNA-dependent mechanisms. Stem Cells Transl Med.

[35] Harting MT, Srivastava AK, Zhaorigetu S, Bair H, Prabhakara KS, Toledano Furman NE (2018). Inflammation-stimulated mesenchymal stromal cell-derived extracellular vesicles attenuate inflammation. Stem Cells.

[36] Shen B, Liu J, Zhang F, Wang Y, Qin Y, Zhou Z (2016). CCR2 positive exosome released by mesenchymal stem cells suppresses macrophage functions and alleviates ischemia/reperfusion-induced renal injury. Stem Cells Int.

[37] Wang J, Jia H, Zhang B, Yin L, Mao F, Yu J (2018). HucMSC exosome-transported 14-3-3zeta prevents the injury of cisplatin to HK-2 cells by inducing autophagy in vitro. Cytotherapy..

[38] Shi S, Zhang Q, Xia Y, You B, Shan Y, Bao L (2016). Mesenchymal stem cell-derived exosomes facilitate nasopharyngeal carcinoma progression. Am J Cancer Res.

[39] Mao J, Liang Z, Zhang B, Yang H, Li X, Fu H (2017). UBR2 enriched in p53 deficient mouse bone marrow mesenchymal stem cell-exosome promoted gastric cancer progression via Wnt/beta-catenin pathway. Stem Cells.

[40] Kitai Y, Kawasaki T, Sueyoshi T, Kobiyama K, Ishii KJ, Zou J (2017). DNA-containing exosomes derived from cancer cells treated with Topotecan activate a STING-dependent pathway and reinforce antitumor immunity. J Immunol.

[41] Shelke GV, Jang SC, Yin Y, Lässer C (2016). Lötvall J.

[42] Chevillet JR, Kang Q, Ruf IK, Briggs HA, Vojtech LN, Hughes SM (2014). Quantitative and stoichiometric analysis of the microRNA content of exosomes. Proc Natl Acad Sci U S A.

[43] Monsel A, Zhu YG, Gennai S, Hao Q, Hu S, Rouby JJ (2015). Therapeutic effects of human mesenchymal stem cell-derived microvesicles in severe pneumonia in mice. Am J Respir Crit Care Med.

[44] Zhu YG, Feng XM, Abbott J, Fang XH, Hao Q, Monsel A (2014). Human mesenchymal stem cell microvesicles for treatment of Escherichia coli endotoxin-induced acute lung injury in mice. Stem Cells.

[45] Hu S, Park J, Liu A, Lee J, Zhang X (2018). Hao Q et al.

[46] Tang XD, Shi L, Monsel A, Li XY, Zhu HL, Zhu YG (2017). Mesenchymal stem cell microvesicles attenuate acute lung injury in mice partly mediated by Ang-1 mRNA. Stem Cells.

[47] Ju GQ (2015). Cheng J, Zhong L, Wu S, Zou XY. Zhang GY et al Microvesicles derived from human umbilical cord mesenchymal stem cells facilitate tubular epithelial cell dedifferentiation and growth via hepatocyte growth factor induction PLoS One.

[48] Tomasoni S, Longaretti L, Rota C, Morigi M, Conti S, Gotti E (2013). Transfer of growth factor receptor mRNA via exosomes unravels the regenerative effect of mesenchymal stem cells. Stem Cells Dev.

[49] Ragni E, Banfi F, Barilani M, Cherubini A, Parazzi V, Larghi P (2017). Extracellular vesicle-shuttled mRNA in mesenchymal stem cell communication. Stem Cells.

[50] McBride JD, Rodriguez-Menocal L, Candanedo A, Guzman W, Garcia-Contreras M (2018). Badiavas EV.

[51] Abdouh M, Hamam D, Gao ZH, Arena V, Arena M, Arena GO (2017). Exosomes isolated from cancer patients' sera transfer malignant traits and confer the same phenotype of primary tumors to oncosuppressor-mutated cells. J Exp Clin Cancer Res.

[52] Lee TH, Chennakrishnaiah S, Meehan B, Montermini L, Garnier D, D'Asti E (2016). Barriers to horizontal cell transformation by extracellular vesicles containing oncogenic H-ras. Oncotarget..

[53] Kumarswamy R, Volkmann I, Thum T (2011). Regulation and function of miRNA-21 in health and disease. RNA Biol.

[54] Cheng X, Zhang G, Zhang L, Hu Y, Zhang K, Sun X (2018). Mesenchymal stem cells deliver exogenous miR-21 via exosomes to inhibit nucleus pulposus cell apoptosis and reduce intervertebral disc degeneration. J Cell Mol Med.

[55] Cui GH, Wu J, Mou FF, Xie WH, Wang FB, Wang QL (2018). Exosomes derived from hypoxia-preconditioned mesenchymal stromal cells ameliorate cognitive decline by rescuing synaptic dysfunction and regulating inflammatory responses in APP/PS1 mice. FASEB J.

[56] Fang S, Xu C, Zhang Y, Xue C, Yang C, Bi H (2016). Umbilical cord-derived mesenchymal stem cell-derived exosomal microRNAs suppress myofibroblast differentiation by inhibiting the transforming growth factor-beta/SMAD2 pathway during wound healing. Stem Cells Transl Med.

[57] Jackson WM, Nesti LJ, Tuan RS (2012). Mesenchymal stem cell therapy for attenuation of scar formation during wound healing. Stem Cell Res Ther.

[58] Shi B, Wang Y, Zhao R, Long X, Deng W, Wang Z (2018). Bone marrow mesenchymal stem cell-derived exosomal miR-21 protects C-kit+ cardiac stem cells from oxidative injury through the PTEN/PI3K/Akt axis. PLoS One.

[59] Wang K, Jiang Z, Webster KA, Chen J, Hu H, Zhou Y (2017). Enhanced cardioprotection by human endometrium mesenchymal stem cells driven by exosomal microRNA-21. Stem Cells Transl Med.

[60] Gras C, Ratuszny D, Hadamitzky C, Zhang H, Blasczyk R, Figueiredo C (2015). miR-145 contributes to hypertrophic scarring of the skin by inducing myofibroblast activity. Mol Med.

[61] Wang YS, Li SH, Guo J, Mihic A, Wu J, Sun L (2014). Role of miR-145 in cardiac myofibroblast differentiation. J Mol Cell Cardiol.

[62] Mei Li-Li, Wang Wen-Jun, Qiu Yun-Tan, Xie Xiu-Feng, Bai Jie, Shi Zhi-Zhou (2017). miR-145-5p Suppresses Tumor Cell Migration, Invasion and Epithelial to Mesenchymal Transition by Regulating the Sp1/NF-κB Signaling Pathway in Esophageal Squamous Cell Carcinoma. International Journal of Molecular Sciences.

[63] Ozen M, Gulluoglu S, Sevli S, Guzel E, Karatas OF, Bayrak OF (2015). Overexpression of miR-145-5p inhibits proliferation of prostate cancer cells and reduces SOX2 expression. Cancer Investig.

[64] Pan Y, Ye C, Tian Q, Yan S, Zeng X, Xiao C (2018). miR-145 suppresses the proliferation, invasion and migration of NSCLC cells by regulating the BAX/BCL-2 ratio and the caspase-3 cascade. Oncol Lett.

[65] Takahara Kiyoshi, Ii Masaaki, Inamoto Teruo, Nakagawa Takatoshi, Ibuki Naokazu, Yoshikawa Yuki, Tsujino Takuya, Uchimoto Taizo, Saito Kenkichi, Takai Tomoaki, Tanda Naoki, Minami Koichiro, Uehara Hirofumi, Komura Kazumasa, Hirano Hajime, Nomi Hayahito, Kiyama Satoshi, Asahi Michio, Azuma Haruhito (2016). microRNA-145 Mediates the Inhibitory Effect of Adipose Tissue-Derived Stromal Cells on Prostate Cancer. Stem Cells and Development.

[66] Qian X, Xu C, Fang S, Zhao P, Wang Y, Liu H (2016). Exosomal microRNAs derived from umbilical mesenchymal stem cells inhibit hepatitis C virus infection. Stem Cells Transl Med.

[67] Kim J, Jiang J, Badawi M, Schmittgen TD (2017). miR-221 regulates CD44 in hepatocellular carcinoma through the PI3K-AKT-mTOR pathway. Biochem Biophys Res Commun.

[68] Yin Z, Xu M, Li P (2017). miRNA-221 acts as an oncogenic role by directly targeting TIMP2 in non-small-cell lung carcinoma. Gene..

[69] Ma M, Chen S, Liu Z, Xie H, Deng H, Shang S (2017). miRNA-221 of exosomes originating from bone marrow mesenchymal stem cells promotes oncogenic activity in gastric cancer. Onco Targets Ther.

[70] Creecy CM, O'Neill CF, Arulanandam BP, Sylvia VL, Navara CS, Bizios R (2013). Mesenchymal stem cell osteodifferentiation in response to alternating electric current. Tissue Eng Part A.

[71] Zimolag E, Borowczyk-Michalowska J, Kedracka-Krok S, Skupien-Rabian B, Karnas E, Lasota S (1864). Electric field as a potential directional cue in homing of bone marrow-derived mesenchymal stem cells to cutaneous wounds. Biochim Biophys Acta.

[72] Abramovitch-Gottlib L, Gross T, Naveh D, Geresh S, Rosenwaks S, Bar I (2005). Low level laser irradiation stimulates osteogenic phenotype of mesenchymal stem cells seeded on a three-dimensional biomatrix. Lasers Med Sci.

[73] Peng F, Wu H, Zheng Y, Xu X, Yu J (2012). The effect of noncoherent red light irradiation on proliferation and osteogenic differentiation of bone marrow mesenchymal stem cells. Lasers Med Sci.

[74] Nurkovic J, Zaletel I, Nurkovic S, Hajrovic S, Mustafic F, Isma J (2017). Combined effects of electromagnetic field and low-level laser increase proliferation and alter the morphology of human adipose tissue-derived mesenchymal stem cells. Lasers Med Sci.

[75] Steward AJ, Kelly DJ (2015). Mechanical regulation of mesenchymal stem cell differentiation. J Anat.

[76] Baraniak PR, McDevitt TC (2012). Scaffold-free culture of mesenchymal stem cell spheroids in suspension preserves multilineage potential. Cell Tissue Res.

[77] Lo YP, Liu YS, Rimando MG, Ho JH, Lin KH, Lee OK (2016). Three-dimensional spherical spatial boundary conditions differentially regulate osteogenic differentiation of mesenchymal stromal cells. Sci Rep.

[78] Kirkham GR, Elliot KJ, Keramane A, Salter DM, Dobson JP, El Haj AJ (2010). Hyperpolarization of human mesenchymal stem cells in response to magnetic force. IEEE Trans Nanobioscience.

[79] Zhang Y, Chopp M, Zhang ZG, Katakowski M, Xin H, Qu C (2017). Systemic administration of cell-free exosomes generated by human bone marrow derived mesenchymal stem cells cultured under 2D and 3D conditions improves functional recovery in rats after traumatic brain injury. Neurochem Int.

[80] Patel DB, Gray KM, Santharam Y, Lamichhane TN, Stroka KM, Jay SM (2017). Impact of cell culture parameters on production and vascularization bioactivity of mesenchymal stem cell-derived extracellular vesicles. Bioeng Transl Med.

[81] Yao Y, Zhang F, Wang L, Zhang G, Wang Z, Chen J (2009). Lipopolysaccharide preconditioning enhances the efficacy of mesenchymal stem cells transplantation in a rat model of acute myocardial infarction. J Biomed Sci.

[82] Wang ZJ, Zhang FM, Wang LS, Yao YW, Zhao Q, Gao X (2009). Lipopolysaccharides can protect mesenchymal stem cells (MSCs) from oxidative stress-induced apoptosis and enhance proliferation of MSCs via toll-like receptor (TLR)-4 and PI3K/Akt. Cell Biol Int.

[83] Duijvestein M, Wildenberg ME, Welling MM, Hennink S, Molendijk I, van Zuylen VL (2011). Pretreatment with interferon-gamma enhances the therapeutic activity of mesenchymal stromal cells in animal models of colitis. Stem Cells.

[84] Ti D, Hao H, Tong C, Liu J, Dong L, Zheng J (2015). LPS-preconditioned mesenchymal stromal cells modify macrophage polarization for resolution of chronic inflammation via exosome-shuttled let-7b. J Transl Med.

[85] Song Y, Dou H, Li X, Zhao X, Li Y, Liu D (2017). Exosomal miR-146a contributes to the enhanced therapeutic efficacy of interleukin-1beta-primed mesenchymal stem cells against Sepsis. Stem Cells.

[86] Zhang Q, Fu L, Liang Y, Guo Z, Wang L, Ma C, et al. Exosomes originating from MSCs stimulated with TGF-beta and IFN-gamma promote Treg differentiation. J Cell Physiol. 2018. 10.1002/jcp.26436 PMC13482522 29336475

[87] Wei X, Du Z, Zhao L, Feng D, Wei G, He Y (2009). IFATS collection: the conditioned media of adipose stromal cells protect against hypoxia-ischemia-induced brain damage in neonatal rats. Stem Cells.

[88] Hashemi SM, Hassan ZM, Pourfathollah AA, Soudi S, Shafiee A, Soleimani M (2013). Comparative immunomodulatory properties of adipose-derived mesenchymal stem cells conditioned media from BALB/c, C57BL/6, and DBA mouse strains. J Cell Biochem.

[89] Lopatina T, Bruno S, Tetta C, Kalinina N, Porta M, Camussi G (2014). Platelet-derived growth factor regulates the secretion of extracellular vesicles by adipose mesenchymal stem cells and enhances their angiogenic potential. Cell Commun Signal.

[90] Wang Y, Lu X, He J, Zhao W (2015). Influence of erythropoietin on microvesicles derived from mesenchymal stem cells protecting renal function of chronic kidney disease. Stem Cell Res Ther.

[91] Zhu J, Lu K, Zhang N, Zhao Y, Ma Q, Shen J, et al. Myocardial reparative functions of exosomes from mesenchymal stem cells are enhanced by hypoxia treatment of the cells via transferring microRNA-210 in an nSMase2-dependent way. Artif Cells Nanomed Biotechnol. 2017:1–12. 10.1080/21691401.2017.1388249 PMC5955787 29141446

[92] Du W, Zhang K, Zhang S, Wang R, Nie Y, Tao H (2017). Enhanced proangiogenic potential of mesenchymal stem cell-derived exosomes stimulated by a nitric oxide releasing polymer. Biomaterials..

[93] Pascucci L, Cocce V, Bonomi A, Ami D, Ceccarelli P, Ciusani E (2014). Paclitaxel is incorporated by mesenchymal stromal cells and released in exosomes that inhibit in vitro tumor growth: a new approach for drug delivery. J Control Release.

[94] Pachler K, Lener T, Streif D, Dunai ZA, Desgeorges A, Feichtner M (2017). A good manufacturing practice-grade standard protocol for exclusively human mesenchymal stromal cell-derived extracellular vesicles. Cytotherapy..

[95] Takeda YS, Xu Q (2015). Neuronal differentiation of human mesenchymal stem cells using exosomes derived from differentiating neuronal cells. PLoS One.

[96] Lozito TP, Tuan RS (2014). Endothelial and cancer cells interact with mesenchymal stem cells via both microparticles and secreted factors. J Cell Mol Med.

[97] Yin Y, Shelke GS, Jang SC, Lässer C, Wennmalm S, Hoffmann HJ et al. Regulation of mesenchymal stem cell function by TGFβ-1 on mast cell extracellular vesicles — role of endosomal retention. bioRxiv. 2017.

[98] Li X, Wang S, Zhu R, Li H, Han Q, Zhao RC (2016). Lung tumor exosomes induce a pro-inflammatory phenotype in mesenchymal stem cells via NFkappaB-TLR signaling pathway. J Hematol Oncol.

[99] Lai RC, Yeo RW, Padmanabhan J, Choo A, de Kleijn DP, Lim SK (2016). Isolation and characterization of exosome from human embryonic stem cell-derived C-Myc-immortalized mesenchymal stem cells. Methods Mol Biol.

[100] Yu B, Kim HW, Gong M, Wang J, Millard RW, Wang Y (2015). Exosomes secreted from GATA-4 overexpressing mesenchymal stem cells serve as a reservoir of anti-apoptotic microRNAs for cardioprotection. Int J Cardiol.

[101] Hnatiuk AP, Ong SG, Olea FD, Locatelli P, Riegler J, Lee WH, et al. Allogeneic mesenchymal stromal cells overexpressing mutant human hypoxia-inducible factor 1-alpha (HIF1-alpha) in an ovine model of acute myocardial infarction. J Am Heart Assoc. 2016;5. 10.1161/JAHA.116.003714 PMC5015403 27385426

[102] Li H, Liu D, Li C, Zhou S, Tian D, Xiao D (2017). Exosomes secreted from mutant-HIF-1alpha-modified bone-marrow-derived mesenchymal stem cells attenuate early steroid-induced avascular necrosis of femoral head in rabbit. Cell Biol Int.

[103] Mirotsou M, Zhang Z, Deb A, Zhang L, Gnecchi M, Noiseux N (2007). Secreted frizzled related protein 2 (Sfrp2) is the key Akt-mesenchymal stem cell-released paracrine factor mediating myocardial survival and repair. Proc Natl Acad Sci U S A.

[104] Ridge SM, Sullivan FJ, Glynn SA (2017). Mesenchymal stem cells: key players in cancer progression. Mol Cancer.

[105] Roccaro AM, Sacco A, Maiso P, Azab AK, Tai YT, Reagan M (2013). BM mesenchymal stromal cell-derived exosomes facilitate multiple myeloma progression. J Clin Invest.

[106] Yuan Z, Kolluri KK, Sage EK, Gowers KH, Janes SM (2015). Mesenchymal stromal cell delivery of full-length tumor necrosis factor-related apoptosis-inducing ligand is superior to soluble type for cancer therapy. Cytotherapy..

[107] Yuan Z, Kolluri KK, Gowers KH, Janes SM (2017). TRAIL delivery by MSC-derived extracellular vesicles is an effective anticancer therapy. J Extracell Vesicles.

[108] Wang B, Yao K, Huuskes BM, Shen HH, Zhuang J, Godson C (2016). Mesenchymal stem cells deliver exogenous microRNA-let7c via exosomes to attenuate renal fibrosis. Mol Ther.

[109] Lou G, Yang Y, Liu F, Ye B, Chen Z, Zheng M (2017). MiR-122 modification enhances the therapeutic efficacy of adipose tissue-derived mesenchymal stem cells against liver fibrosis. J Cell Mol Med.

[110] Lou G, Song X, Yang F, Wu S, Wang J, Chen Z (2015). Exosomes derived from miR-122-modified adipose tissue-derived MSCs increase chemosensitivity of hepatocellular carcinoma. J Hematol Oncol.

[111] Sharif S, Ghahremani MH, Soleimani M (2018). Delivery of exogenous miR-124 to glioblastoma multiform cells by Wharton’s jelly mesenchymal stem cells decreases cell proliferation and migration, and confers chemosensitivity. Stem Cell Rev.

[112] Katakowski M, Buller B, Zheng X, Lu Y, Rogers T, Osobamiro O (2013). Exosomes from marrow stromal cells expressing miR-146b inhibit glioma growth. Cancer Lett.

[113] Zhang Y, Chopp M, Liu XS, Katakowski M, Wang X, Tian X (2017). Exosomes derived from mesenchymal stromal cells promote axonal growth of cortical neurons. Mol Neurobiol.

[114] Shen H, Yao X, Li H, Li X, Zhang T, Sun Q (2018). Role of exosomes derived from miR-133b modified MSCs in an experimental rat model of intracerebral hemorrhage. J Mol Neurosci.

[115] Chen L, Lu FB, Chen DZ, Wu JL, Hu ED, Xu LM (2018). BMSCs-derived miR-223-containing exosomes contribute to liver protection in experimental autoimmune hepatitis. Mol Immunol.

[116] Tao SC, Yuan T, Zhang YL, Yin WJ, Guo SC, Zhang CQ (2017). Exosomes derived from miR-140-5p-overexpressing human synovial mesenchymal stem cells enhance cartilage tissue regeneration and prevent osteoarthritis of the knee in a rat model. Theranostics..

[117] Tao SC, Guo SC, Li M, Ke QF, Guo YP, Zhang CQ (2017). Chitosan wound dressings incorporating exosomes derived from microRNA-126-overexpressing synovium mesenchymal stem cells provide sustained release of exosomes and heal full-thickness skin defects in a diabetic rat model. Stem Cells Transl Med.

[118] Mendt M, Kamerkar S, Sugimoto H, McAndrews KM, Wu CC, Gagea M, et al. Generation and testing of clinical-grade exosomes for pancreatic cancer. JCI Insight. 2018;3. 10.1172/jci.insight.99263 PMC5931131 29669940

[119] Kotmakci M, Bozok CV (2015). Extracellular vesicles as natural nanosized delivery systems for small-molecule drugs and genetic material: steps towards the future nanomedicines. J Pharm Pharm Sci.

[120] Galipeau J (2013). The mesenchymal stromal cells dilemma--does a negative phase III trial of random donor mesenchymal stromal cells in steroid-resistant graft-versus-host disease represent a death knell or a bump in the road?. Cytotherapy..

[121] Capasso S, Alessio N, Squillaro T, Di Bernardo G, Melone MA, Cipollaro M (2015). Changes in autophagy, proteasome activity and metabolism to determine a specific signature for acute and chronic senescent mesenchymal stromal cells. Oncotarget..

[122] Liang X, Zhang L, Wang S, Han Q, Zhao RC (2016). Exosomes secreted by mesenchymal stem cells promote endothelial cell angiogenesis by transferring miR-125a. J Cell Sci.

[123] Pakravan Katayoon, Babashah Sadegh, Sadeghizadeh Majid, Mowla Seyed Javad, Mossahebi-Mohammadi Majid, Ataei Farangis, Dana Nasim, Javan Mohammad (2017). MicroRNA-100 shuttled by mesenchymal stem cell-derived exosomes suppresses in vitro angiogenesis through modulating the mTOR/HIF-1α/VEGF signaling axis in breast cancer cells. Cellular Oncology.

[124] Mayourian J, Ceholski DK, Gorski PA, Mathiyalagan P, Murphy JF, Salazar SI (2018). Exosomal microRNA-21-5p mediates mesenchymal stem cell paracrine effects on human cardiac tissue contractility. Circ Res.

[125] Ono M., Kosaka N., Tominaga N., Yoshioka Y., Takeshita F., Takahashi R.-u., Yoshida M., Tsuda H., Tamura K., Ochiya T. (2014). Exosomes from bone marrow mesenchymal stem cells contain a microRNA that promotes dormancy in metastatic breast cancer cells. Science Signaling.

[126] Barrera-Ramirez J, Lavoie JR, Maganti HB, Stanford WL, Ito C, Sabloff M (2017). Micro-RNA profiling of exosomes from marrow-derived mesenchymal stromal cells in patients with acute myeloid leukemia: implications in leukemogenesis. Stem Cell Rev.

[127] Nakamura Y, Miyaki S, Ishitobi H, Matsuyama S, Nakasa T, Kamei N (2015). Mesenchymal-stem-cell-derived exosomes accelerate skeletal muscle regeneration. FEBS Lett.

[128] Bruno S, Tapparo M, Collino F, Chiabotto G, Deregibus MC, Soares Lindoso R (2017). Renal regenerative potential of different extracellular vesicle populations derived from bone marrow mesenchymal stromal cells. Tissue Eng Part A..

[129] Figueroa J, Phillips LM, Shahar T, Hossain A, Gumin J, Kim H (2017). Exosomes from glioma-associated mesenchymal stem cells increase the tumorigenicity of glioma stem-like cells via transfer of miR-1587. Cancer Res.

[130] Joerger-Messerli MS, Oppliger B, Spinelli M, Thomi G, di Salvo I, Schneider P (2018). Extracellular vesicles derived from Wharton’s jelly mesenchymal stem cells prevent and resolve programmed cell death mediated by perinatal hypoxia-ischemia in neuronal cells. Cell Transplant.

[131] Wu XQ, Yan TZ, Wang ZW, Wu X, Cao GH, Zhang C (2017). BM-MSCs-derived microvesicles promote allogeneic kidney graft survival through enhancing micro-146a expression of dendritic cells. Immunol Lett.

[132] Chen C, Wang D, Moshaverinia A, Liu D, Kou X, Yu W (2017). Mesenchymal stem cell transplantation in tight-skin mice identifies miR-151-5p as a therapeutic target for systemic sclerosis. Cell Res.

[133] Lee JK, Park SR, Jung BK, Jeon YK, Lee YS, Kim MK (2013). Exosomes derived from mesenchymal stem cells suppress angiogenesis by down-regulating VEGF expression in breast cancer cells. PLoS One.

[134] Kulkarni R, Bajaj M, Ghode S, Jalnapurkar S, Limaye L, Kale VP (2018). Intercellular transfer of microvesicles from young mesenchymal stromal cells rejuvenates aged murine hematopoietic stem cells. Stem Cells.

[135] Gangadaran P, Rajendran RL, Lee HW, Kalimuthu S, Hong CM, Jeong SY (2017). Extracellular vesicles from mesenchymal stem cells activates VEGF receptors and accelerates recovery of hindlimb ischemia. J Control Release.

[136] Feng Y, Huang W, Wani M, Yu X, Ashraf M (2014). Ischemic preconditioning potentiates the protective effect of stem cells through secretion of exosomes by targeting Mecp2 via miR-22. PLoS One.

[137] Wang X, Gu H, Qin D, Yang L, Huang W, Essandoh K (2015). Exosomal miR-223 contributes to mesenchymal stem cell-elicited cardioprotection in polymicrobial sepsis. Sci Rep.

[138] Gong M, Yu B, Wang J, Wang Y, Liu M, Paul C (2017). Mesenchymal stem cells release exosomes that transfer miRNAs to endothelial cells and promote angiogenesis. Oncotarget..

[139] Zhu L. L., Huang X., Yu W., Chen H., Chen Y., Dai Y. T. (2017). Transplantation of adipose tissue-derived stem cell-derived exosomes ameliorates erectile function in diabetic rats. Andrologia.

[140] Wang Y, Fu B, Sun X, Li D, Huang Q, Zhao W (2015). Differentially expressed microRNAs in bone marrow mesenchymal stem cell-derived microvesicles in young and older rats and their effect on tumor growth factor-beta1-mediated epithelial-mesenchymal transition in HK2 cells. Stem Cell Res Ther.

[141] Xin H, Li Y, Liu Z, Wang X, Shang X, Cui Y (2013). MiR-133b promotes neural plasticity and functional recovery after treatment of stroke with multipotent mesenchymal stromal cells in rats via transfer of exosome-enriched extracellular particles. Stem Cells.

[142] Xin H, Li Y, Buller B, Katakowski M, Zhang Y, Wang X (2012). Exosome-mediated transfer of miR-133b from multipotent mesenchymal stromal cells to neural cells contributes to neurite outgrowth. Stem Cells.

[143] Yang Jia, Zhou Cheng-Zhi, Zhu Rui, Fan Heng, Liu Xing-Xing, Duan Xue-Yun, Tang Qing, Shou Zhe-Xing, Zuo Dong-Mei (2017). miR-200b-containing microvesicles attenuate experimental colitis associated intestinal fibrosis by inhibiting epithelial-mesenchymal transition. Journal of Gastroenterology and Hepatology.

[144] Lang FM, Hossain A, Gumin J, Momin EN, Shimizu Y, Ledbetter D (2018). Mesenchymal stem cells as natural biofactories for exosomes carrying miR-124a in the treatment of gliomas. Neuro-Oncology.

[145] O'Brien KP, Khan S, Gilligan KE, Zafar H, Lalor P, Glynn C (2018). Employing mesenchymal stem cells to support tumor-targeted delivery of extracellular vesicle (EV)-encapsulated microRNA-379. Oncogene..

[146] Li X, Liu L, Yang J, Yu Y, Chai J, Wang L (2016). Exosome derived from human umbilical cord mesenchymal stem cell mediates MiR-181c attenuating burn-induced excessive inflammation. EBioMedicine..

[147] Qu Y, Zhang Q, Cai X, Li F, Ma Z, Xu M (2017). Exosomes derived from miR-181-5p-modified adipose-derived mesenchymal stem cells prevent liver fibrosis via autophagy activation. J Cell Mol Med.

[148] Chen Y, Zhao Y, Chen W, Xie L, Zhao ZA, Yang J (2017). MicroRNA-133 overexpression promotes the therapeutic efficacy of mesenchymal stem cells on acute myocardial infarction. Stem Cell Res Ther.

[149] Yu B, Gong M, Wang Y, Millard RW, Pasha Z, Yang Y (2013). Cardiomyocyte protection by GATA-4 gene engineered mesenchymal stem cells is partially mediated by translocation of miR-221 in microvesicles. PLoS One.

